# A new integrated genetic and transcriptomic approach for investigating *DUX4* and *DUX4C*

**DOI:** 10.1038/s10038-025-01450-x

**Published:** 2026-01-16

**Authors:** Zhaohui Zhuang, Mahoko Takahashi Ueda, Kensuke Yamaguchi, Yuta Kochi

**Affiliations:** 1 Department of Genomic Function and Diversity, Medical Research Laboratory, Institute of Integrated Research, Institute of Science Tokyo, Tokyo, Japan; 2 Biomedical Engineering Research Innovation Center, Laboratory for Biomaterials and Bioengineering, Institute of Integrated Research, Institute of Science Tokyo, Tokyo, Japan

**Keywords:** Genome, Gene expression

## Abstract

The double homeobox 4 gene (*DUX4*) and its centromeric paralogue, *DUX4C*, reside in the subtelomeric region of chromosome 4 and have been implicated in facioscapulohumeral muscular dystrophy (FSHD) and cancers. However, the high sequence similarity between these genes, together with the widespread presence of *DUX4*-like paralogues across the human genome, has hindered accurate genotyping and expression profiling using short-read sequencing. To elucidate the genetic architecture and potential disease-associated functions of *DUX4* and *DUX4C*, we first identified two distinct *DUX4C* haplotypes with expression quantitative trait effects—*DUX4C*-4qα and *DUX4C*-4qβ. We then integrated them with known *DUX4* haplotypes to generate a reference genome, D4Ref-T2T, using long-read sequencing. Haplotype analysis indicated strong linkage disequilibrium between *DUX4C* and *DUX4* haplotypes (*r*^2^ = 0.86). We further characterized full-length *DUX4C* mRNA isoforms and established a corresponding transcriptome reference. Applying these resources to breast tumor, FSHD, and lymphoblastoid cell line datasets revealed that *DUX4* is expressed in both breast tumor and FSHD tissues, whereas *DUX4C* shows expression only in breast tumors. Differential gene expression and Gene Ontology enrichment analyses further suggested that *DUX4C* expression is associated with activation of pathways involved in leukocyte differentiation, chemotaxis, and cell migration.

## Introduction

The double homeobox 4 (*DUX4*) gene resides in the subtelomeric region of human chromosome 4, embedded within a macrosatellite array composed of approximately 1–100 copies of 3.3-kb D4Z4 tandem repeats. When the number of repeats contracts to ≤10 and a functional polyadenylation signal (PAS) is present within the terminal unit, mature *DUX4* transcripts are produced, enabling expression of full-length *DUX4* protein, which has been implicated in facioscapulohumeral muscular dystrophy (FSHD) and several cancer types [1, 2].

The complex structure of the *DUX4* locus has been redefined in the recent telomere-to-telomere (T2T) project [3], and upstream of the macrosatellite array lies an additional repeat in the T2T-CHM13v2.0 genome. This repeat encodes the centromeric paralogue *DUX4C* (also known as *DUX4L9*), which has also been associated with FSHD pathology [4]. However, the extremely high sequence similarity between *DUX4* and *DUX4C*, together with incomplete annotation of *DUX4C* transcripts, has made it difficult to distinguish these genes using short-read sequencing. These limitations have hindered efforts to define *DUX4C*-specific functions and to clarify its relationship with the telomeric gene *DUX4* (hereafter *DUX4T*).

To overcome these challenges, we combined genomic and transcriptomic analyses using both short- and long-read sequencing technologies. This integrative framework aimed to: (i) delineate the major haplotypes of *DUX4C* and *DUX4T* through systematic analysis of publicly available short-read datasets and prior literature; (ii) reconstruct complete haplotype sequences using long-read whole-genome (LWGS) data to generate a haplotype-resolved reference suitable for accurate genotyping from short-read whole-genome (SWGS) data, while also evaluating the genetic relationship between *DUX4C* and *DUX4T*; and (iii) characterize transcript diversity using long-read RNA sequencing to develop an expanded transcript reference. Leveraging these resources, we further investigated the roles and regulatory mechanisms of *DUX4C* and *DUX4T* across multiple biological contexts, including breast tumors, FSHD, and interferon-α2 (IFNα2)–stimulated lymphoblastoid cell lines (LCLs).

## Materials and methods

### DNA and RNA sample preparation

Japanese LCLs, derived from B lymphocytes, were obtained from the NIGMS Human Genetic Cell Repository at the Coriell Institute. Cells were cultured in RPMI-1640 medium supplemented with 15% fetal bovine serum and 1% penicillin–streptomycin. For stimulation experiments, LCLs were treated with 50 ng/mL recombinant human IFNα2 (BioLegend) for 6 h, while untreated cells served as controls. Approximately 1 × 10⁷ cells were collected for each extraction.

Genomic DNA was isolated using the DNeasy Blood & Tissue Kit (QIAGEN), and total RNA was extracted using the RNeasy Mini Kit (QIAGEN), following the manufacturer’s instructions. Nucleic acid concentration and purity were assessed with a NanoDrop spectrophotometer (Thermo Fisher Scientific), and RNA integrity was evaluated using an Agilent 2100 Bioanalyzer (RNA 6000 Nano Kit). Only RNA samples with RNA integrity number (RIN) > 9.0 were used for downstream analyses.

### Nanopore long-read sequencing

Genomic DNA libraries from LCLs (NA18943 and NA18948) were prepared using the SQK-LSK109 ligation sequencing kit (Oxford Nanopore Technologies) and sequenced on a PromethION platform equipped with FLO-PRO001 flow cells according to the manufacturer’s guidelines. Basecalling was performed using Guppy v6.4.1 in super-accurate mode.

For long-read transcriptome sequencing, cDNA libraries from IFNα2-stimulated NA18943 were prepared using the SQK-PCB114.24 cDNA-PCR Barcoding Kit V14 (Oxford Nanopore Technologies) and sequenced on PromethION PRO114M flow cells. Basecalling was performed using Dorado v0.9.8 in super-accurate mode.

### Sequencing data acquisition

RNA-seq datasets for FSHD and control samples—including LCLs (n = 18), myoblasts (n = 6), and myotubes (n = 6)—were retrieved from Gene Expression Omnibus (GEO) accession GSE153523 [5]. Breast tumor and normal tissue RNA-seq data were obtained from GEO accession GSE233242, including Luminal A (n = 29), Luminal B (n = 3), triple-negative breast cancer (TNBC, n = 9), HER2-positive tumors (n = 2), and normal tissue controls (n = 43) [6].

SWGS data from Japanese LCLs (n = 104) were obtained from the 1000 Genomes Project for *DUX4C* and *DUX4T* genotyping. RNA-seq data from a subset of these Japanese LCLs, including IFNα2-stimulated (n = 94) and non-stimulated (n = 20) samples, were retrieved from the DDBJ Sequence Read Archive (SRA; accession DRA016395) [7]. Long-read RNA-seq data generated in this study have been deposited in the DDBJ Sequence Read Archive (DRA) under BioProject accession number PRJDB39925.

### Haplotype analysis of *DUX4C* region

Expression quantitative trait loci (eQTL) data for *DUX4L9* (ENSG00000224807.5) in LCLs were obtained from the GTEx Analysis Release V10 (dbGaP accession phs000424.v10.p2) on 08/01/2025. Variants with a normalized effect size (NES) ≥ 0.5 were selected (20 variants in total). Corresponding genotypes for these variants in the European (n = 373) and Japanese (n = 94) populations were obtained from the 1000 Genomes Project [8]. Variants with a minor allele frequency (MAF) < 0.1 were excluded. Pairwise linkage disequilibrium (LD) was calculated using PLINK v1.90b6.16 [9], and LD heatmaps were visualized in Python using Seaborn. Haplotype phasing and frequency estimation were performed using Beagle v5.5 [10].

LWGS data from NA18943 and NA18948 were basecalled using Guppy and aligned to the GRCh38 reference genome with Minimap2 v2.30 under default settings [11]. DNA methylation states were inferred using DeepMod2 v0.3.0 with the r9.4.1 model [12]. For each variant, mean methylation levels were calculated within a ± 50 bp window centered on the position.

### Simulated mapping of *DUX4T* breakpoint sequences

Simulated short-read datasets were generated from known *DUX4T* haplotype breakpoint sequences (Table S2a) using Sandy v0.24 with the parameters -c 30 -m 50 -e 0.01 -t single-end. Simulated reads were aligned to the CHM13v2.0 and GRCh38 reference genomes using BWA-MEM v0.7.19 with default parameters [13]. Alignments were sorted, and low-quality reads (MAPQ < 10) were filtered using Samtools v1.22.1.

### Extraction and alignment of *DUX4*-like genes

*DUX4*-like (*DUX4L*) sequences in the CHM13v2.0 reference genome were identified by extracting regions annotated as “*DUX4*” from both the genome FASTA and corresponding GFF3 files. Coding regions were compared against canonical *DUX4* domains defined in the *DUX4* mRNA reference (NCBI protein accession: NP_149418.2). Based on the presence or absence of key domains, *DUX4L* sequences were classified into three groups: (i) those containing both homeobox domains (HD1 and HD2) and the transcriptional activation domain (TAD); (ii) those retaining HD1 and HD2 but lacking a TAD; and (iii) those lacking all three domains. Multiple sequence alignment was conducted using ApE v3.1.8.1 [14].

### Acquisition of complete *DUX4C* and *DUX4T* haplotype sequences

We used LWGS data selected from Japanese LCL samples, including one homozygous sample for *DUX4C*-4qα and *DUX4T*-4qB [15], as well as another homozygous sample for *DUX4C*-4qβ and *DUX4T*-4qA, to extract the complete *DUX4* sequences. The *DUX4C* sequence within the *FRG1*–*DUX4L9*–*FRG2* region (chr4:193,282,466–193,399,128) was retrieved from the CHM13v2.0 T2T genome. *DUX4T* haplotype sequences were obtained from the NCBI Nucleotide Database, including *DUX4T*-4A161 (HM190178.1), *DUX4T*-10A166 (HM190186.1), and *DUX4T*-4B163 (HM190161.1). Because *DUX4T*-4qB and *DUX4T*-10qB share identical breakpoint sequences, making them indistinguishable for genotyping, we extracted a complete *DUX4T*-B sequence from NA18943 (*DUX4T*: 4qB/4qB; 10qA/10qA), which served as the reference sequence for both 4qB and 10qB *DUX4T* haplotypes.

From these sequences, 200-bp regions spanning the *DUX4T* breakpoints and adjacent 3′ segments were extracted as mapping references [15]. LWGS data from NA18943 and NA18948 were aligned using Winnowmap2 v2.03 with a k-mer size of 20 [16]. Reads with MAPQ < 10 were removed using Samtools. Breakpoint-containing reads corresponding to *DUX4T*-4qA (NA18948), *DUX4T*-10qA (NA18948), and *DUX4T*-B (NA18943) were identified, and 21,680-bp regions spanning the last two D4Z4 repeats through exon 7 of the *DUX4* long isoform (GenBank: NR_137167.1) were extracted. D4Z4 tandem repeat arrays (chr4:193,282,466–193,558,261 and chr10:134,615,120–134,741,148 in CHM13v2.0) were masked, and the three extracted 21,680-bp segments, together with the *DUX4C* sequence, were integrated into the masked genome to construct a custom reference.

To refine haplotype accuracy, LWGS reads from NA18943 and NA18948 were re-aligned to the custom reference with Winnowmap2, followed by variant calling using PEPPER-Margin-DeepVariant v0.8 with default settings [17]. Variants passing quality thresholds were incorporated to generate finalized haplotype-resolved sequences: *DUX4C*-4qα, *DUX4C*-4qβ, *DUX4T*-4qA, *DUX4T*-10qA, and *DUX4T*-B.

### D4Ref-T2T genome construction and sequencing data alignment

Sequencing-error–corrected 15-kb haplotype sequences for *DUX4C*-4qα, *DUX4C*-4qβ, *DUX4T*-4qA, *DUX4T*-10qA, and *DUX4T*-B were incorporated into the CHM13v2.0 genome after masking the D4Z4 tandem repeat arrays and endogenous *DUX4* loci (chr4:193,376,884–193,391,884; chr4:193,395,464–193,555,598; chr10:134,615,120–134,738,536). This produced a custom genotyping reference, designated D4Ref-T2T. To validate the completeness and accuracy of each haplotype sequence, LWGS reads from NA18943 and NA18948 were aligned to D4Ref-T2T using Winnowmap2 (k-mer size 20). Reads with MAPQ < 10 were removed using Samtools, and alignments were inspected in IGV v2.19.7 [18].

The D4Ref-T2T genome was then indexed and used as the reference for SWGS alignment. Reads were mapped using BWA-MEM implemented in Parabricks v4.3.2-1 under default settings, followed by MAPQ-based filtering (MAPQ < 10) using Samtools.

### Genotyping of *DUX4C* and *DUX4T* haplotypes

Haplotype-specific motifs were defined for both genes. For *DUX4C*, two 6-bp sequences spanning rs7696384 and rs7696390 in the 3′ UTR were used to distinguish the *DUX4C*-4qα and *DUX4C*-4qβ haplotypes. For *DUX4T*, the canonical 6-bp PAS motif was used to identify *DUX4T*-4qA, and analogous 6-bp sequences at syntenic positions were used to genotype *DUX4T*-10qA and *DUX4T*-B.

Coverage of each haplotype-specific motif was calculated as the mean read depth across the six nucleotide positions:

$${{\rm{S}}}{{\rm{pecific}}}\; {{\rm{Motif}}}\; {{\rm{Coverage}}}=\frac{{\sum}_{{{\rm{i}}}=1}^{6}\,\,{{{\rm{motif}}}\,{{\rm{base}}}\; {{\rm{depth}}}}_{{{\rm{i}}}}}{6}$$

Genotypes were inferred by computing the relative proportion of each haplotype-specific motif. For *DUX4C*:

$${{{\rm{Proportion}}}}_{{DUX}4{C\_}4q\alpha }=\frac{{{{\rm{Coverage}}}}_{{DUX}4{C\_}4{{\rm{q}}}{{\rm{\alpha }}}}}{{{{\rm{Coverage}}}}_{{DUX}4{C\_}4{{\rm{q}}}{{\rm{\alpha }}}}+{{{\rm{Coverage}}}}_{{DUX}4{C\_}4{{\rm{q}}}{{\rm{\beta }}}}}$$

and for *DUX4T*:

$${{{\rm{Proportion}}}}_{{DUX}4{T\_}4{qA}}=\frac{{{{\rm{Coverage}}}}_{{DUX}4{T\_}4{{\rm{qA}}}}}{{{{\rm{Coverage}}}}_{{DUX}4{T\_}4{{\rm{qA}}}}+{{{\rm{Coverage}}}}_{{DUX}4{T\_}10{{\rm{qA}}}}+{{{\rm{Coverage}}}}_{{DUX}4{T\_}{{\rm{B}}}}}$$

$${{{\rm{Proportion}}}}_{{DUX}4{T\_}10{qA}}=\frac{{{{\rm{Coverage}}}}_{{DUX}4{T\_}10{{\rm{qA}}}}}{{{{\rm{Coverage}}}}_{{DUX}4{T\_}4{{\rm{qA}}}}+{{{\rm{Coverage}}}}_{{DUX}4T\_10{{\rm{qA}}}}+{{{\rm{Coverage}}}}_{{DUX}4{T\_}{{\rm{B}}}}}$$

Expected motif proportions for *DUX4C* genotypes are 1 for 4qα/4qα, 0.5 for 4qα/4qβ, and 0 for 4qβ/4qβ. Because experimental variation can shift observed values away from these theoretical ratios, we applied permissive thresholds: 0.75–1.0 for 4qα/4qα, 0.25–0.75 for 4qα/4qβ, and 0–0.25 for 4qβ/4qβ.

For chromosome 4 *DUX4T*, theoretical proportions are 0.5 for 4qA/4qA, 0.25 for 4qA/4qB, and 0 for 4qB/4qB. Practical threshold ranges were set as ≥0.375 for 4qA/4qA, 0.125–0.375 for 4qA/4qB, and 0–0.125 for 4qB/4qB. For chromosome 10 *DUX4T*, the same threshold scheme (≥0.375, 0.125–0.375, and 0–0.125) was used to classify 10qA/10qA, 10qA/10qB, and 10qB/10qB genotypes, respectively. Haplotype phasing, frequency estimation, and pairwise LD calculations were performed using Beagle.

### Novel *DUX4C* isoform identification

Short-read RNA-seq data from Japanese LCLs stimulated with IFN-α2 for 6 h were aligned to the D4Ref-T2T reference genome using STAR v2.7.11b [19], with the following parameters: --outFilterMultimapNmax 10, --outFilterIntronMotifs None, --outSJfilterDistToOtherSJmin 0 0 0 0, and --twopassMode Basic.

Genome annotation was derived from the CHM13v2.0 GTF file but modified such that the *DUX4L9* locus was updated to reflect *DUX4C*-4qα coordinates (12,742–14,000). Long-read RNA-seq data from IFNα2-stimulated NA18943 were mapped using FLAIR v2.2.0. Splice-junction correction was applied with flair correct using STAR-generated junctions (SJ.out.tab) and the modified GTF. Isoforms were reconstructed with flair collapse using the parameters --support 2, --filter comprehensive, and --no_gtf_end_adjustment, based on the same annotation [20]. Coding sequences and predicted amino acid sequences were generated with SQANTI3 v5.5.1 [21]. Candidate *DUX4C* isoforms were curated using the following criteria: a 5′ UTR longer than 50 bp; a FANTOM5 CAGE peak located within the 5′ UTR; an initiating methionine (M) codon; and the presence of a Kozak consensus sequence at the translation start site.

### *DUX4* expression, differential gene expression, and gene ontology enrichment analyses

The longer *DUX4C* isoform (*DUX4C*-v1) was added to the transcriptome reference (D4Trans-hg38), which was constructed on GENCODE Release 49 (GRCh38.p14) [22]. Short-read RNA-seq data were aligned to D4Trans-hg38 and quantified with Salmon v1.10.2 [23]. Transcript-level estimates from quant.sf files were converted to gene-level values, and all entries annotated as “*DUX4*” were combined to represent *DUX4T* expression using the comprehensive GTF annotation. Expression levels were normalized as transcripts per million (TPM), and differential expression analysis of *DUX4C* and *DUX4T* was conducted: breast tumors versus matched controls (n = 43 each), FSHD patients versus controls (n = 15 each), and IFNα2-stimulated LCLs versus unstimulated controls (n = 20 each). Statistical significance was assessed using the Mann–Whitney U test implemented in pandas and scipy.stats. Data visualization was carried out using seaborn and matplotlib.

Counts per million (CPM) for the *DUX4C*-v1 exon 2 region were obtained from samtools-derived coverage. Gene-level count matrices were imported from Salmon outputs using tximport in R. Differential gene expression (DEG) analysis was conducted with DESeq2 on IFNα2-stimulated LCLs, grouped into case samples (*DUX4C*: 4qα/4qα; *DUX4T*: 4qB/4qB; CPM > 1; n = 3) and control samples (*DUX4C*: 4qβ/4qβ; *DUX4T*: 4qA/4qA; CPM = 0; n = 3) [24]. Control samples were selected from the candidate samples (n = 9) at random using a custom Python script. Genes encoding immunoglobulins (*IGH*, *IGK*, and *IGL*) were removed before Gene Ontology (GO) enrichment. GO enrichment was performed with clusterProfiler using padj < 0.05 and log₂ fold change > 0.5 as thresholds. Enrichment results were processed and visualized with ggplot2 and dplyr, with annotations supplied by org.Hs.eg.db.

### Ethics

This study was approved by the Ethics Committee of the Institute of Science, Tokyo (Approved No.: O2019-005-08).

## Results

### *DUX4C* haplotypes in human populations

**Fig. 1:**
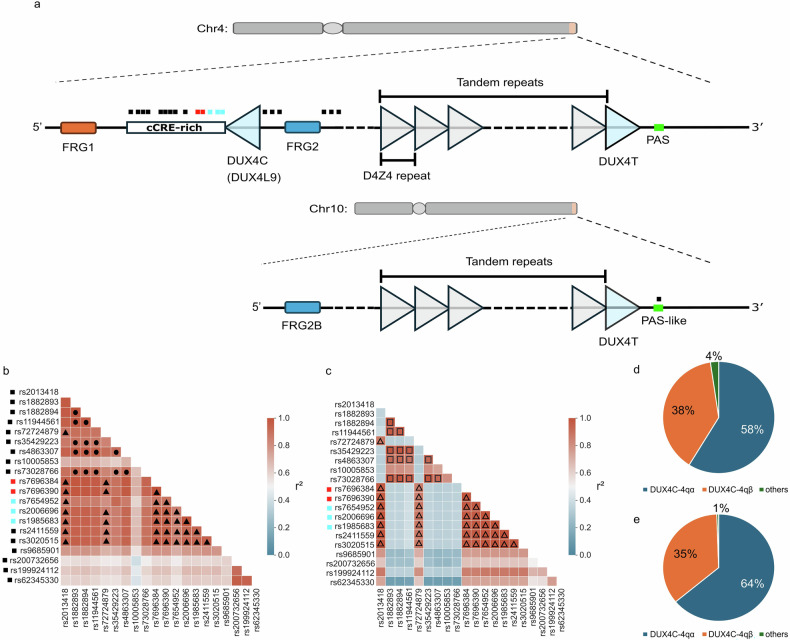
Genomic loci and haplotype architecture of *DUX4C* and *DUX4T*. **a** *DUX4C* (*DUX4L9*) is located between *FRG1* and *FRG2* on chromosome 4, whereas *DUX4T* resides within the terminal D4Z4 repeats on chromosomes 4 and 10. SNPs are shown as colored dots; *DUX4C*-defining SNPs are indicated in red (genotyping SNPs) and blue. SNPs located in the 3′ region of *DUX4C* correspond to cCREs. PAS and PAS-like motifs of *DUX4T* are shown as green bars, and SNP in *DUX4*-10qA is shown as black dot. **b** Pairwise LD (r^2^) matrix of *DUX4L9* eQTLs in the European population. The same SNPs and color scheme as in (**a**) are used to indicate functional annotation. Symbols “●” and “▲” denote block 1 and block 2, respectively. **c** Pairwise LD (r^2^) matrix of *DUX4L9* eQTLs in the Japanese population. Symbols “□” and “△” denote block 1 and block 2, respectively. **d** Haplotype frequency distribution of *DUX4C*-4qα and *DUX4C*-4qβ in the European population. **e** Haplotype frequency distribution of *DUX4C*-4qα and *DUX4C*-4qβ in the Japanese population

To distinguish *DUX4* paralogues, the centromeric *DUX4* repeat on chromosome 4 was defined as *DUX4C*, the terminal *DUX4* repeats on chromosomes 4 and 10 were classified collectively as *DUX4T*, and the intervening array was designated as the D4Z4 tandem repeats (Fig. 1a). The GTEx project previously identified multiple single-nucleotide polymorphisms (SNPs) acting as eQTLs for *DUX4C* (*DUX4L9*) in LCLs from European individuals. To explore the underlying genetic structure of this locus, we examined haplotype organization across populations. eQTLs with NES ≥ 0.5 were selected from GTEx (20 variants; Fig. 1a; Table S1a). LD patterns in European and Japanese populations revealed two distinct haplotype blocks in each population as grouped by the LD index (r^2^ > 0.8) (Fig. 1b, c; Table S1e, g).

The 3′ region of *DUX4C* is enriched for cis-regulatory elements (cCREs) across multiple tissues and cell types, as annotated by ENCODE [25], and exhibits hypomethylation in LCLs (methylation rate < 0.2; Table S1b, c). Based on these features, we selected five eQTL SNPs with potential regulatory relevance (rs7696384, rs7696390, rs7654952, rs2006696, and rs1985683). These variants showed strong LD (r^2^ > 0.95) in both populations and were suitable markers for defining functional haplotypes. Haplotype inference using these variants identified two major haplotypes—T-A-T-A-G and G-G-G-G-C—corresponding to *DUX4C*-4qα and *DUX4C*-4qβ, respectively. Their frequencies were similar between populations (*DUX4C*-4qα: 58% in Europeans, 64% in Japanese; *DUX4C*-4qβ: 38% in Europeans, 35% in Japanese) (Fig. 1d, e; Table S1d, f), supporting their use as robust genetic markers for the *DUX4C* locus. The *DUX4C* haplotype sequences in the GRCh38 and CHM13v2.0 assemblies are identical except for their SNP alleles, with GRCh38 containing *DUX4C*‑4qβ and CHM13v2.0 containing *DUX4C*‑4qα.

### Comparative analysis of *DUX4T* haplotypes integrated in reference genomes

**Fig. 2:**
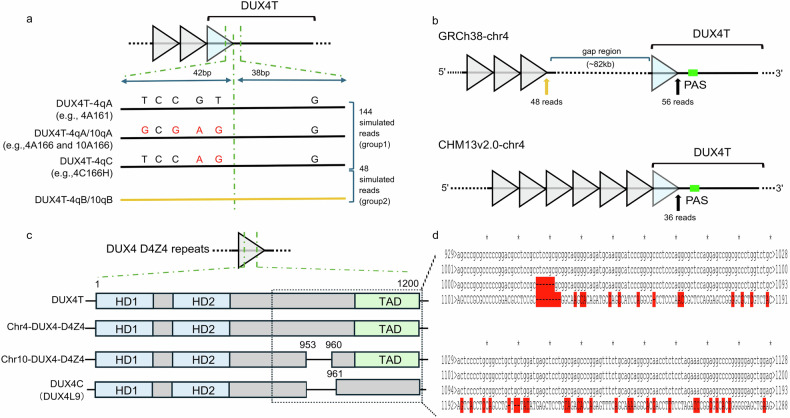
Comparison of GRCh38 and CHM13v2.0 assemblies and *DUX4* family genes. **a** Breakpoint sequences for *DUX4T* haplotypes, including a 42-bp repeat and 38-bp flanking sequence. SNPs relative to *DUX4T*-4qA161 are indicated in red. Breakpoint sequences of *DUX4T*-4qB and *DUX4T*-10qB, highlighted in yellow, show substantial divergence from those of *DUX4T*-4qA, *DUX4T*-10qA, and *DUX4T*-4qC. Simulated single-end short reads were generated separately for group 1 and group 2 sequences. **b** Simulated reads mapped to GRCh38 and CHM13v2.0, with group 1 shown in black and group 2 in yellow. **c** Comparative sequences of *DUX4* family genes annotated in the CHM13v2.0 assembly. **d** Detailed alignment of *DUX4* family genes (from top to bottom: *DUX4T*; chr4-*DUX4*-D4Z4; chr10-*DUX4*-D4Z4; and *DUX4C* (*DUX4L9*)). Variants relative to *DUX4T* are shown in red

*DUX4T* exhibits multiple haplotypes, among which *DUX4T*-4qA has been linked to disease susceptibility [2, 15]. In the GRCh38 reference genome, an assembly gap of approximately 82 kb is present downstream of the D4Z4 tandem repeats and upstream of *DUX4T* on chromosome 4 (Fig. 2b). This missing sequence was later resolved in the CHM13v2.0 assembly using long-read sequencing.

To evaluate the accuracy of these assemblies, we performed simulated mapping with *DUX4T*-specific breakpoint sequences. Eighty–base pair junction sequences spanning the transition between the final D4Z4 repeat and the 3′ flanking region—unique to each *DUX4T* haplotype [15]—were used to generate simulated single-end short reads, which were aligned to both GRCh38 and CHM13v2.0 (Fig. 2a, b; Table S2a).

Mapping results showed that in GRCh38, 56 reads corresponding to group 1 haplotypes (4qA, 4qC, and 10qA) aligned to the *DUX4T* region (chr4:190,175,561–190,175,579; 3′ side of the assembly gap), whereas 48 reads from group 2 haplotypes (4qB and 10qB) mapped to the final D4Z4 repeat at the 5′ side of the gap (Fig. 2b; Table S2b, c). In contrast, in CHM13v2.0, 36 group 1 reads aligned precisely to chr4:193,543,366–193,543,392 (Table S2d), whereas group 2 reads failed to map. These findings suggest that GRCh38 likely mis-integrated *DUX4T*-4qA and *DUX4T*-4qB haplotypes on chromosome 4, whereas CHM13v2.0 accurately represents the *DUX4T*-4qA haplotype alone, providing a more precise and haplotype-resolved assembly.

### Comparative analysis of *DUX4*-like paralogues in CHM13v2.0 genome

*DUX4*-like paralogues (*DUX4Ls*) are dispersed across multiple chromosomes, with intact open reading frames (ORFs) restricted to the D4Z4 tandem repeats on chromosomes 4 and 10 (Fig. 1) [26]. Due to their high sequence similarity to *DUX4C* and *DUX4T*, these paralogues can complicate genotyping and isoform identification, especially when using short-read sequencing data. To elucidate their genetic characteristics, we extracted 96 *DUX4*-like sequences from the CHM13v2.0 assembly for comparative analysis.

Sequence alignment revealed that *DUX4Ls* embedded within the D4Z4 arrays on chromosomes 4 and 10—together with *DUX4T*—retain intact ORFs encompassing both HD1 and HD2 and TADs (Fig. 2c). In contrast, *DUX4Ls* located outside the D4Z4 regions (e.g., on chromosomes 9 and 18) harbor disruptive SNPs within their coding sequences, resulting in frameshifts or premature stop codons. The sole exception was *DUX4C* (annotated as *DUX4L9*), which retains both HDs but lacks the C-terminal TAD (Fig. 2c, d). These observations indicate that, in the CHM13v2.0 genome, only *DUX4*-like genes embedded within the D4Z4 tandem repeat arrays maintain full-length ORFs, whereas *DUX4C* contains an ORF that lacks the canonical TAD sequence observed in *DUX4T*. In contrast, *DUX4*-like loci located outside these arrays have become pseudogenized through accumulated mutations.

### Genotyping of *DUX4C* and *DUX4T* using the D4Ref-T2T reference

To establish a comprehensive reference for *DUX4* genotyping, we first extracted complete haplotype sequences from LWGS data of NA18943 (*DUX4C*: 4qα/4qα; *DUX4T*: 4qB/4qB; 10qA/10qA) and NA18948 (*DUX4C*: 4qβ/4qβ; *DUX4T*: 4qA/4qA; 10qA/10qA). Consensus haplotype sequences were generated by applying sample-specific variants to the raw haplotypes (*DUX4C*-4qα, *DUX4T*-4qB, and *DUX4T*-10qA from NA18943; *DUX4C*-4qβ and *DUX4T*-4qA from NA18948). Haplotype-specific SNPs were validated for *DUX4C*, and breakpoint sequences were confirmed for *DUX4T* within each complete haplotype. The 80-bp sequences (breakpoint) of *DUX4C*-4qα and *DUX4C*-4qβ, spanning from the repeat region to the 3′ region, were extracted (Table S2e). Simulated reads were generated from these sequences and aligned to the GRCh38 and CHM13v2.0 assemblies using the same strategy as for *DUX4T*. In both assemblies, reads from both haplotypes mapped to a single locus corresponding to the *DUX4C* locus (Table S2f–i).

**Fig. 3:**
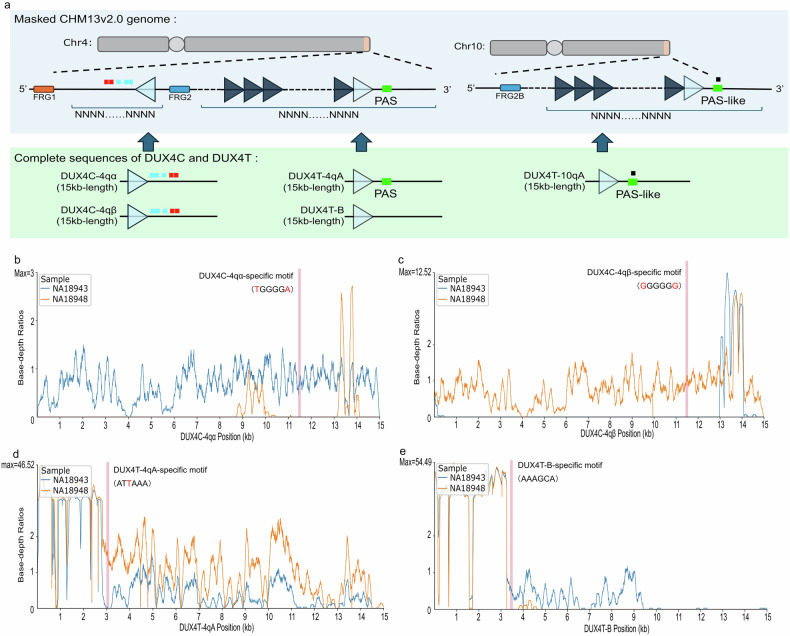
D4Ref-T2T genome architecture and relative base-depth at *DUX4* haplotypes. **a** *DUX4* and D4Z4 tandem repeat regions in CHM13v2.0 were masked, and complete *DUX4C* and *DUX4T* haplotype sequences were inserted as accessory contigs. **b**–**e** Relative base-depths calculated from SWGS data of NA18943 and NA18948. Haplotype-specific motifs are indicated by red lines. Panels show mapping results for **b** *DUX4C*-4qα, **c** *DUX4C*-4qβ, **d** *DUX4T*-4qA, and **e** *DUX4T*-B

To minimize ambiguous alignment of reads derived from *DUX4C* and *DUX4T* to the highly repetitive D4Z4 tandem arrays, all *DUX4L* sequences within the D4Z4 arrays on chromosomes 4 and 10 were masked in the CHM13v2.0 assembly. Verified haplotype sequences were then incorporated as accessory contigs, generating a *DUX4*-specific telomere-to-telomere reference genome, termed D4Ref-T2T (Fig. 3a). Remapping of NA18943 and NA18948 LWGS reads confirmed complete haplotype coverage consistent with the known genotypes of both samples (Fig. S1).

A genotyping framework for *DUX4C* and *DUX4T* was established using SWGS data aligned to D4Ref-T2T. Relative base-depth—defined as per-base coverage normalized to the genome-wide average—was used to assess mapping specificity, with values approaching 1 indicating uniquely mapped, non-redundant alignments. Haplotype-informative positions included SNPs distinguishing *DUX4C*-4qα from *DUX4C*-4qβ, the functional PAS specific to *DUX4T*-4qA, and corresponding 6-bp sequences at syntenic positions of *DUX4T*-10qA and *DUX4T*-B, all located within high-specificity regions (Fig. 3b–e).

Genotypes were inferred by calculating the proportion of each haplotype-specific motif relative to the summed coverage of all motifs (Table S3a, b). In the Japanese cohort, D4Ref-T2T–based genotypes of *DUX4T* matched those from the previous study using simple sequence length polymorphism analysis and Southern blotting [15] in 41 of 45 cases. Among the four unmatched samples, we classified NA18951 and NA19005 as 4qB/4qB rather than 4qA/4qB, based on our results. This decision was supported by the observed proportions of the *DUX4T*-4qA motif, which were 0 and 0.02 (a single read), respectively. The single 4qA read was manually inspected and determined to be a mismatched read originating from 4qB, indicating a 4qB/4qB genotype. In NA19003, the presence of *DUX4T*-10qB may have biased the result of 4qB. *DUX4T* genotyping of chromosome 10 identified two samples—including NA19003—classified as 10qA/10qB. In NA19003, the proportions of *DUX4T*-4qA (0.38) and *DUX4T*-10qA (0.31) supported assignment as 4qA/4qA and 10qA/10qB genotypes, which were inconsistent with the previous result of 4qA/4qB and 10qA/10qA. The remaining sample, NA18995, was classified as 4qA/4qA and 10qA/10qA, as both *DUX4T*-4qA and *DUX4T*-10qA showed high proportions (0.38 and 0.40). This made it difficult to assign the relatively lower proportion of the *DUX4T*-B allele (0.23) to either 4qB or 10qB, although its genotype in the previous study was 4qA/4qB and 10qA/10qA. Due to the ambiguous genotyping results observed in NA19003 and NA18995, we excluded these samples, along with two additionally genotyped samples (NA19002 and NA19009), from the subsequent analyses, as they exhibited high proportions of *DUX4T*-4qA (≥0.375) and *DUX4T*-B (≥0.05), which may have led to misclassification of 4qA/4qB as 4qA/4qA.

### Detection of *DUX4C* haplotype-derived isoforms and structural characterization

**Fig. 4:**
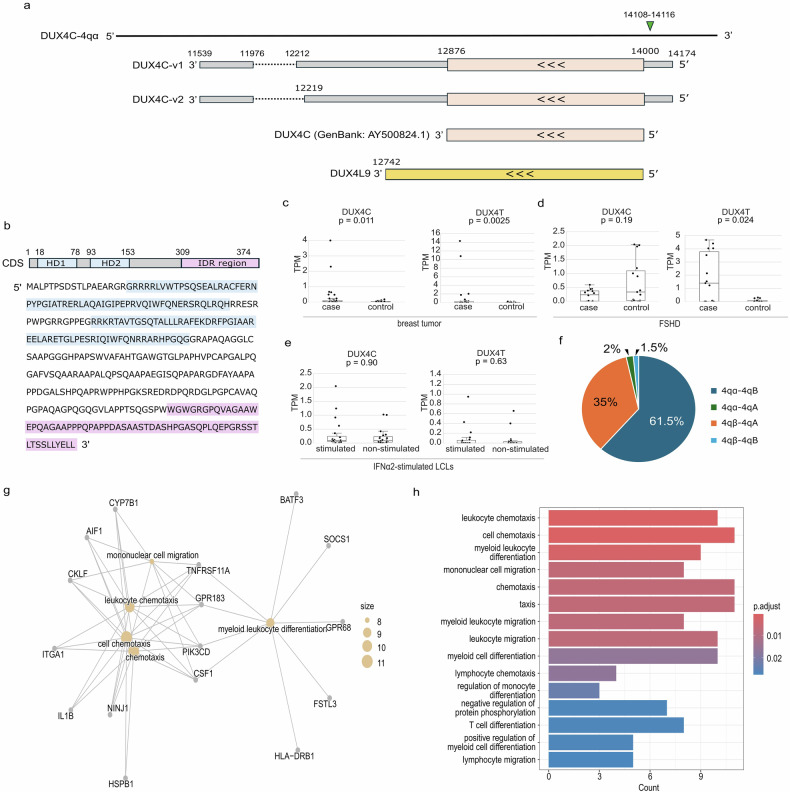
Novel *DUX4C* isoforms, expression patterns of *DUX4C* and *DUX4T*, and functional analysis of *DUX4C*. **a** Novel isoforms *DUX4C*-v1 and *DUX4C*-v2 compared with previously published *DUX4C* and *DUX4L9* transcripts. The CAGE-seq peak indicating the transcription start site is shown as a green triangle. **b** Domain architecture of *DUX4C* isoforms. HD1 and HD2 are shown in blue, and the IDR is shown in pink. Expression of *DUX4C* and *DUX4T* in different sample contexts: **c** breast tumor (43 cases and 43 controls), **d** FSHD patients (15 cases and 15 controls), and **e** IFNα2-stimulated LCLs (20 stimulated and 20 non-stimulated). Statistical significance was assessed using the Mann–Whitney U test (p < 0.05). **f** Haplotype frequency for *DUX4C* and *DUX4T* in the Japanese population. **g**, **h** GO enrichment analysis of DEGs between IFNα2-stimulated LCLs expressing *DUX4C* (genotype: *DUX4C* 4qα/4qα; *DUX4T* 4qB/4qB, n = 3) and those lacking *DUX4C* expression (genotype: *DUX4C*: 4qβ/4qβ; *DUX4T*: 4qA/4qA, n = 3). Biological Process (BP) of (**g**) cnetplot: gray nodes represent DEGs, orange nodes represent pathways, and orange node size reflects the number of associated DEGs. **h** Barplot: bars indicate enriched GO terms, color represents p-value, and length indicates the number of enriched DEGs

We hypothesized that novel *DUX4C* isoforms are expressed in IFNα2-stimulated LCLs. Long-read RNA-seq data from NA18943 and NA18948 were aligned to the D4Ref-T2T genome, revealing that *DUX4C* isoforms were detected only in NA18943. Isoform prediction for *DUX4C* was therefore performed using a combination of short-read and long-read RNA-seq data from a single IFNα2-stimulated LCL sample (NA18943). After filtering, two novel *DUX4C* isoforms with intact coding sequences were identified and compared with the previously reported *DUX4C* transcript (GenBank: AY500824.1) and *DUX4L9* annotated in the CHM13v2.0 genome (Fig. 4a). The predicted isoforms contained a defined 5′ untranslated region (UTR; *DUX4C*-4qα: 14,000–14,174) and 3′ UTR (11,539–12,876), as well as two splice junctions at positions 11,976–12,212 and 11,976–12,219. In contrast, the previously reported *DUX4C* transcript and the *DUX4L9* annotation lacked recorded 5′ and 3′ UTR structures. These features represent the first complete transcript structure reported for *DUX4C*, and the isoforms were confirmed to originate from the *DUX4C*-4qα haplotype.

We then analyzed the predicted protein domain architecture of *DUX4C* (*DUX4C*-v1). The sequence aligned closely with the previously reported *DUX4C* mRNA (GenBank: AY500824.1) (Fig. 4a). While *DUX4T* harbors a C-terminal TAD, a structured domain essential for transcriptional activity, *DUX4C* lacks sequence conservation in this region. Instead, the C-terminus of *DUX4C* contains an intrinsically disordered region (IDR) (Fig. 4b). To identify potential interaction sites within this region, we used MoRFchibi to predict short Molecular Recognition Features (MoRFs)—binding-prone segments often found in intrinsically disordered sequences [27, 28]. A cluster of high-propensity MoRFs spanning residues 310 to the C-terminus was identified (MCW > 0.7) (Table S4a). We further used Eukaryotic Linear Motif (ELM) predictions to explore functional motifs potentially involved in signaling and regulation mediated by IDR. This analysis revealed several motifs within *DUX4C* IDR, including those associated with signal transduction (e.g., SH2 and PDZ-binding motifs), post-translational modifications (e.g., CK1 and GSK3 phosphorylation sites), and membrane trafficking (e.g., endocytic sorting signals) (Table S4b). These features suggest that the *DUX4C* IDR may function as a dynamic regulatory hub mediating protein–protein interactions and cellular signaling, although additional protein–protein interaction assays will be required to validate this possibility.

### Expression dynamics of *DUX4C* and *DUX4T* across biological contexts, and a chemotactic role for *DUX4C*

To investigate potential functions of *DUX4C*, we constructed a customized transcriptome reference, D4Trans-hg38, by updating GENCODE Release 49 (GRCh38.p14) to replace the *DUX4L9* entry with the full-length *DUX4C*-v1 transcript identified from our long-read RNA-seq data. Short-read RNA-seq datasets from diverse biological contexts—including human breast tumors (n = 43), FSHD patient samples (n = 15), and IFNα2-stimulated LCLs (n = 20)—were quantified against D4Trans-hg38. Both *DUX4C* and *DUX4T* were significantly upregulated in breast tumor tissues relative to normal breast samples (Fig. 4c). In contrast, only *DUX4T* exhibited significant upregulation in FSHD patient tissues, while *DUX4C* expression remained unchanged (Fig. 4d). Neither gene showed significant expression changes upon IFNα2 stimulation in LCLs (Fig. 4e). Interestingly, five samples in the FSHD control (unaffected family members of FSHD patients) exhibited elevated *DUX4C* expression (TPM > 1), suggesting that *DUX4C* may exert weaker myotoxic effects or potentially serve tissue-protective or reparative roles compared with *DUX4T*.

To further explore the relationship between genetic background and *DUX4C* function, we analyzed D4Ref-T2T–based haplotypes of *DUX4C* and *DUX4T* in the Japanese cohort (n = 100). We identified 123 4qα-4qB and 70 4qβ–4qA haplotypes, accounting for 61.5% and 35% of all haplotypes, respectively (Fig. 4f; Table S3c), where a strong LD was observed between these two loci (*r*^2^ = 0.86). These findings suggest a potential linkage between *DUX4C*-4qα and *DUX4T*-4qB, indicating that *DUX4C* expression may be modulated by *DUX4T* at the genetic level, possibly functioning in a compensatory or modulatory manner.

Given the potential haplotype effects of *DUX4C*, IFNα2-stimulated LCLs were stratified into case (*DUX4C*: 4qα/4qα; *DUX4T*: 4qB/4qB; CPM > 1; n = 3) and control (*DUX4C*: 4qβ/4qβ; *DUX4T*: 4qA/4qA; CPM = 0; n = 3) (Table S5a) for DEG and GO enrichment analyses, providing a limited yet exploratory assessment of *DUX4C* function. Upregulated genes (Table S5b) in the case (high-*DUX4C* expressing LCLs) were significantly enriched in pathways related to immune-related chemotactic processes, including “leukocyte chemotaxis,” “cell chemotaxis,” and “lymphocyte chemotaxis” (Fig. 4g, h; Table S5c), suggesting that *DUX4C* may modulate immune cell recruitment or migration in a haplotype-specific manner. This functional association is further supported by ELM-predicted linear motifs within the C-terminal IDR of *DUX4C*, which include interaction domains such as SH2, PDZ, and phosphorylation sites. These motifs are commonly found in signaling proteins involved in immune pathways, raising the possibility that *DUX4C* indirectly contributes to chemotactic signaling through dynamic protein–protein interactions or post-translational modifications.

## Discussion

In this study, we developed a *DUX4*-specific reference genome (D4Ref-T2T) and transcriptome (D4Trans-hg38), enabling accurate genotyping and isoform identification of *DUX4C* and *DUX4T*. Comparison of genotyping results between CHM13v2.0 and D4Ref-T2T indicated that both references can reliably resolve *DUX4C* genotypes. In contrast, *DUX4T*-4qB lacks a complete reference sequence in CHM13v2.0 and diverges substantially from the *DUX4T*-4qA sequence represented. D4Ref-T2T addresses this limitation by providing a comprehensive reference that supports large-scale *DUX4T* genotyping using SWGS data.

A limitation of the current D4Ref-T2T genotyping framework is the observed discrepancy in four samples relative to previous reports, suggesting that PCR validation with *DUX4T*-specific primers may be necessary for definitive genotyping. Additionally, as previous studies have demonstrated that *DUX4T* expression is regulated by methylation within the D4Z4 tandem repeats [2], *DUX4C* expression may similarly be affected by local methylation. This underscores the importance of evaluating D4Z4 repeat structures to elucidate the functional roles of *DUX4* haplotypes. However, evaluating these effects remains technically challenging: SWGS reads are substantially shorter than the ~3.3-kb D4Z4 repeat unit, and even with D4Ref-T2T, multi-mapping ambiguity persists within the repeat region (Fig. 3b–e). Consequently, investigating DNA methylation—particularly in a phased context—and its regulatory impact on *DUX4C* represents a critical avenue for future research.

Beyond establishing a genotyping framework, we identified previously uncharacterized full-length *DUX4C* isoforms in LCLs. Because *DUX4C* isoforms were determined from a single sample (NA18943), this analysis is limited in its ability to establish haplotype associations, and additional functional isoforms may remain undiscovered. We observed that in short-read RNA-seq data from IFNα2-stimulated LCL controls, a subset of samples with the *DUX4C*-4qβ/4qβ genotype exhibited low but detectable *DUX4C* expression (TPM < 0.15; Table S6b), suggesting the possible presence of *DUX4C* transcripts derived from the *DUX4C*-4qβ haplotype. However, in the absence of long-read RNA-seq data, the presence and structure of these transcripts could not be conclusively validated. In contrast, samples with the *DUX4C*: 4qα/4qα genotype consistently showed higher *DUX4C* expression levels (TPM ~ 2.1), with a statistically significant difference compared with the 4qβ/4qβ group (Mann–Whitney U test, p < 0.05; Table S6a–c). Taken together, these results suggest that the *DUX4C*-4qα haplotype may enhance *DUX4C* expression, rather than acting as a strict determinant of transcriptional activity.

Although *DUX4C* expression may be a consequence of a specific cellular state or other regulatory factors, we discuss the potential roles of *DUX4C* in signaling pathways. Functional analyses suggest that *DUX4C* harbors multiple phosphorylation-related motifs within its C-terminal region, including CK1 and GSK3β sites. These motifs are associated with signaling modules involved in β-catenin degradation and NFκB activation [29], both of which are central regulators of immune responses. Along with chemotaxis-related gene enrichment in *DUX4C*-expressing LCLs, these features suggest that *DUX4C* may promote immune activation. This contrasts with *DUX4T*, which suppresses interferon-stimulated gene expression [30]. Their opposing functions and distinct expression patterns point to haplotype structure as a potential mechanism balancing *DUX4C* and *DUX4T* activity.

Indeed, haplotype analysis of *DUX4C* and *DUX4T* revealed a strong LD between these two loci (*r*^2^ = 0.86) (Table S3c), although the subtelomeric regions of the genome are known to be susceptible to recombination. Two predominant haplotypes—4qα–4qB and 4qβ–4qA—accounted for 97% of all haplotype combinations. This skewed distribution raises the possibility that negative selection acts against the 4qα–4qA haplotype, which expresses both *DUX4C* and functional *DUX4T*, as well as against 4qβ–4qB, which expresses neither. As *DUX4T* is expressed during the early pre-implantation stage of human embryogenesis [31], the mutually exclusive expression of *DUX4C* and *DUX4T*, governed by two functional haplotypes, may play pivotal roles in human embryonic development.

In summary, our work establishes a framework for analyzing genes located in subtelomeric repeat regions and elucidates a potential mechanistic relationship between *DUX4C* and *DUX4T* in regulating immune activity. These findings lay the groundwork for future studies integrating genomic structure, methylation patterns, and transcript isoform diversity to better understand *DUX4*-related immune phenotypes.

## Supplementary information

Table S1

Table S2

Table S3

Table S4

Table S5

Table S6

Figure S1

Data S1
