# Supplementary material for: A new integrated genetic and transcriptomic approach for investigating DUX4 and DUX4C
Source: J Hum Genet. 2026 Jan 16;71(6):373–81. doi: 10.1038/s10038-025-01450-x (PMC13199055; doi:10.1038/s10038-025-01450-x)
Supplement: Supplementary file 7 — Figure S1 [file 10038_2025_1450_MOESM7_ESM.pdf]

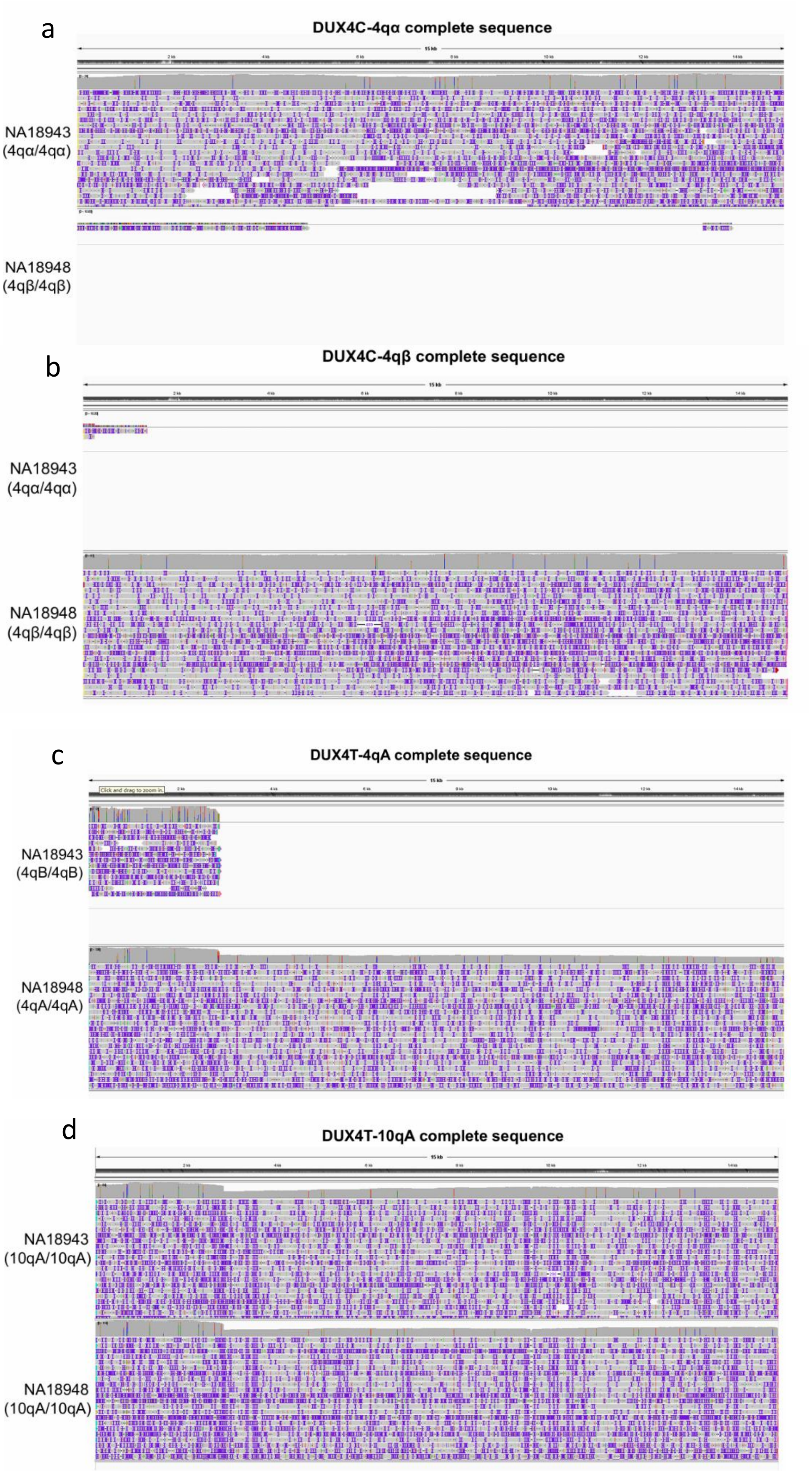
a
DUX4C-4qα complete sequence
NA18943
(4qα/4qα)
NA18948
(4qβ/4qβ)
b
DUX4C-4qβ complete sequence
NA18943
(4qα/4qα)
NA18948
(4qβ/4qβ)
c
DUX4T-4qA complete sequence
Click and drag to zoom in.
NA18943
(4qB/4qB)
NA18948
(4qA/4qA)
d
DUX4T-10qA complete sequence
NA18943
(10qA/10qA)
NA18948
(10qA/10qA)

e

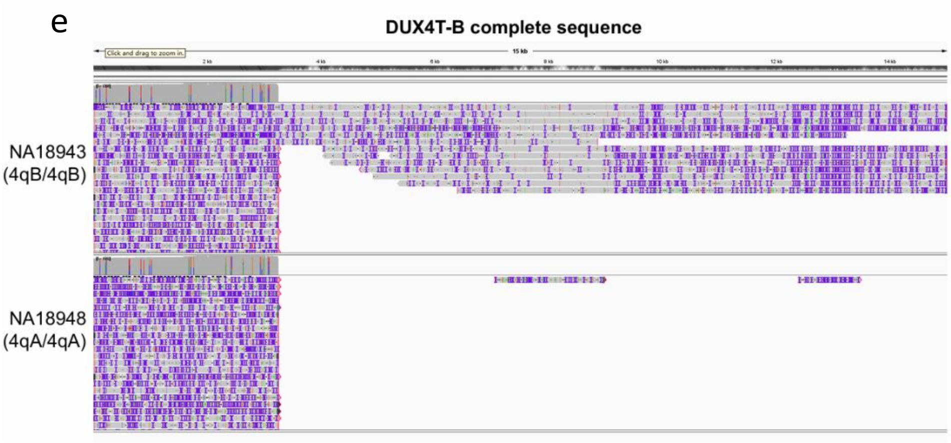

Supplementary Figure 1. LWGS alignments of samples NA18943 and NA18948 to the D4Ref-T2T reference genome

(a) Alignment to the DUX4C-4qα

(b) Alignment to the DUX4C-4qβ

(c) Alignment to the DUX4T-4qA

(d) Alignment to the DUX4T-10qA

(e) Alignment to the DUX4T-B
